# Psychometric properties and validation of the Opening Minds Stigma Scale for Health Care Providers in Slovenia

**DOI:** 10.3389/fpsyt.2025.1671589

**Published:** 2025-11-12

**Authors:** Dorottya Őri, Ana Mirkovic, Polona Rus Prelog

**Affiliations:** 1 Semmelweis University, Institute of Behavioral Sciences, Budapest, Hungary; 2 Department of Mental Health, Heim Pal National Pediatric Institute, Budapest, Hungary; 3 Child Psychiatry Unit, University Children’s Hospital, University Medical Centre Ljubljana, Ljubljana, Slovenia; 4 Centre for Clinical Psychiatry, University Psychiatric Clinic Ljubljana, Ljubljana, Slovenia; 5 Medical Faculty, University of Ljubljana, Ljubljana, Slovenia

**Keywords:** Opening Minds Stigma Scale for Health Care Providers (OMS-HC), mental health-related stigma, psychometric validation, bifactor model, Slovenia, healthcare professionals, stigma assessment, cross-cultural adaptation

## Abstract

**Background:**

Mental health-related stigma among healthcare professionals is a well-documented global concern, contributing to delayed help-seeking, suboptimal treatment adherence, and poorer patient outcomes. In Slovenia, despite growing public and policy efforts to reduce stigma, no validated instrument existed to measure such attitudes among healthcare providers.

**Objective:**

We aimed to explore the psychometric properties of the Slovenian version of the Opening Minds Stigma Scale for Health Care Providers (OMS-HC).

**Methods:**

A diverse sample of 280 Slovenian healthcare professionals completed the OMS-HC. Confirmatory and exploratory factor analyses were used to assess the structure of the scale, and reliability was examined through model-based indices, internal consistency, and test–retest reliability. Convergent validity was evaluated using the MICA-4 scale.

**Results:**

Initial confirmatory factor analysis indicated relatively poor model fit for the original 15-item, three-factor model. Subsequent exploratory and confirmatory factor analyses supported the use of either a 12- or 14-item version. Both demonstrated strong general factor reliability (OmegaH > 0.69; ECV ≈ 0.60), with the 12-item version offering slightly better model fit, while the 14-item version retained broader conceptual content. Test–retest reliability was good for the total score and good to moderate for the subscales. Moderate positive correlations with the MICA-4 scale confirmed convergent validity.

**Conclusions:**

The Slovenian OMS-HC shows good psychometric properties in both its 12- and 14-item formats and is suitable for assessing stigma among healthcare professionals. We recommend the 12-item version for research contexts where parsimony is prioritized, and the 14-item version when broader clinical coverage is needed. Its validation addresses an important methodological gap in Slovenia and provides a reliable tool for stigma monitoring and intervention planning.

## 1 Introduction

Stigmatization of individuals with mental health problems by healthcare professionals is a well-documented global concern, associated with a range of adverse outcomes, including delayed help-seeking, lower treatment adherence, reduced quality of care, and poorer physical and psychological health among affected individuals (1–3). Stigma within healthcare settings may also contribute to self-stigmatization, undermining patients’ self-esteem, recovery, and trust in the healthcare system (4). Given its broad impact, assessing and addressing stigma among healthcare providers is critical for improving mental health outcomes and the overall quality of care.

While stigma reduction has increasingly become a public health priority in many countries, validated measurement tools remain scarce in some regions, including Slovenia. Despite advances such as the Mental Health Act and National Mental Health Programme, and growing multisectoral efforts, implementation is hindered by workforce challenges and lack of standard tools for assessing provider attitudes (5, 6). Community-based multidisciplinary teams—now considered best practice in mental health care—have only recently been promoted as part of this effort, with evidence indicating that such teams improve service user satisfaction, adherence to treatment, social integration, and access to care while reducing stigma (6). Despite these national efforts, scientific research on mental health stigma in Slovenia remains limited. A large multinational study involving Slovenian people with schizophrenia found stigma levels comparable to other European countries, though slightly higher discrimination was reported in employment, family life and social benefits. In contrast, lower discrimination was reported in the education, parenting, and personal safety domains (7).

A few studies focused on healthcare professionals in Slovenia. Nurses were found to have limited knowledge of postnatal mental health (8), while another study highlighted the role of education and ethics in shaping attitudes toward people with mental illness (9). Among medical students, attitudes were generally positive, particularly in final-year students, and an intervention study showed that the Transitions mental health literacy program effectively reduced stigma (10, 11). Similar patterns have been observed in neighboring Central and South-Eastern European countries. A recent multicentric study across five South-Eastern European nations found that stigma toward people with mental illness remained present among medical students, though clinical experience in psychiatry and greater exposure to mental health education significantly reduced stigmatizing attitudes and social distance (12). Similarly, research from the Czech Republic and Slovakia showed that students considering specialization in psychiatry expressed less stigma, particularly in medical schools emphasizing education in psychiatry (13). In Hungary, psychiatrists reported generally positive attitudes toward individuals with mental illness, with direct clinical work linked to lower stigma levels (14).

The Opening Minds Stigma Scale for Health Care Providers (OMS-HC) is a self-reported measurement that was developed to measure stigmatizing attitudes of healthcare professionals toward people with mental illness. Originally consisting of 20 items (15), the scale was revised to a 15-item version with a three-factor structure: Attitude, Disclosure and Help-Seeking, and Social Distance (16). This updated version has since been widely adopted and validated in several countries, including Canada (16), Italy (17), Singapore (18), Chile (19), Hungary (20), Germany (21), Brazil (22), Mexico (23), and Portugal (24). While most studies supported the original three-factor model, others applied more advanced techniques such as bifactor modeling and exploratory structural equation modeling (ESEM), which allow for a more precise evaluation of hierarchical structures. Notably, a large-scale European validation study across 32 countries identified a bifactor ESEM model as the best-fitting solution for the scale, supporting the use of total scores over the subscale scores (25).

To date, Slovenia has only validated one stigma-related tool—the Mental Health Literacy Scale, which focuses on the general population and is not specifically designed for healthcare providers (26). Thus, despite recent policy initiatives and educational interventions, Slovenia lacks a robust, validated instrument to monitor stigma among healthcare professionals, hindering systematic evaluation of stigma-reduction programs and curriculum development. Given the central role of healthcare professionals in Slovenian mental health care and the lack of robust tools for monitoring stigma, locally validated OMS-HC is essential to enable assessment, support training and curriculum development, and inform national anti-stigma efforts. Moreover, national validation is essential because cultural context, language, and healthcare practices can significantly shape stigma-related attitudes; adaptation and validation ensure that the instrument accurately reflects local perspectives, enable relevant comparison across populations, and provide meaningful guidance for intervention design.

The present study aims to address this gap by validating the Slovenian version of the OMS-HC. Specifically, the study investigates its factor structure relative to the original model, evaluates internal consistency and model-based reliability, and assesses test–retest reliability as well as convergent validity.

## 2 Materials and methods

### 2.1 Study design and population

This study represents the Slovenian national continuation of a larger European research project on stigmatizing attitudes of healthcare workers toward people with mental health conditions (preregistration details on ClinicalTrial.gov NCT04644978) (27). It employed an online cross-sectional survey conducted from February 05, 2024, to July 03, 2024, complemented by a longitudinal component to assess test–retest reliability through two rounds of data collection. Ethical approval was obtained as part of the broader European study led by a Hungarian research team and granted by the Regional and Institutional Committee of Science and Research Ethics at Semmelweis University, Budapest, Hungary (SE-RKEB: 189/2019). Participation was voluntary, and informed consent was obtained electronically from all participants via the survey platform. The study was conducted anonymously, with pseudonymization employed to match responses across time points for the test-retest reliability analysis. The survey was distributed online through professional email lists (including psychiatrists, child and adolescent psychiatrists, clinical psychologists, psychiatry and child psychiatry residents, medical students, outpatient clinics, and psychiatric hospitals), and personal invitations. This approach aimed to ensure representativeness across different genders, age groups, and professional backgrounds. The study population included general adult psychiatrists, child and adolescent psychiatrists, clinical psychologists, medical students, and mental health nurses, as well as a group of ,,other” healthcare providers working in mental health settings, comprising occupational therapists, special needs teachers, and social workers. Medical students were included as they comprise a key part of the future mental health workforce and undergo extensive clinical training that significantly shapes their attitudes toward mental illness; their inclusion allows assessment of stigma at early professional stages and supports evaluation of stigma reduction educational programs. The survey design required complete responses before submission, resulting in no missing data. Participants who declined to provide informed consent were automatically led to the end of the survey and thus excluded from the study.

### 2.2 Measurements

#### 2.2.1 Opening minds stigma scale for health care providers

The scale consists of 15 statements reflecting feelings, thoughts, and beliefs about individuals with mental health problems, each rated on a 5-point ordinal scale (16). Respondents who strongly agree with a statement receive a score of 5, while those who strongly disagree receive a score of 1. For items 2, 6, 7, 8, and 14, the scoring is reversed, with strong agreement scored as 1 and strong disagreement as 5 points. The overall stigma score is obtained by summing all item scores, yielding a range from 15 to 75 points, where higher scores reflect greater stigmatizing attitudes. Additionally, scores can be calculated for three subscales: Attitude (6–30 points), Disclosure and Help-Seeking (4–20 points), and Social Distance (5–25 points), with higher subscale scores also indicating stronger stigma.

The English version of the OMS-HC was translated into Slovenian by a psychiatrist proficient in the English language. This forward translation was followed by a translation back into English by another clinician proficient in English. A third healthcare professional checked the back-translation against the original English source, and then an iterative procedure was used to resolve the discrepancies between the original and the back-translated versions of the scale. The concept check was then performed by a focus group of three psychiatrists. For the final Slovenian version of the OMS-HC, see Supplement 1. No formal involvement of service users or people with lived experience was performed, as there are currently no structured service user organizations dedicated to research participation in Slovenia.

#### 2.2.2 Mental illness: clinician’s attitudes-4

We selected the MICA-4 scale to assess convergent validity (28). Like the OMS-HC, it is a self-report instrument designed to measure attitudes toward people with mental health conditions among healthcare professionals. The scale consists of 16 items, producing a total score that ranges from 16 to 96. The scale was translated by two colleagues in psychiatry proficient in English.

### 2.3 Statistical analysis

Sample size (n) and percentages (%) were used to describe demographic data. Confirmatory factor analysis (CFA) was applied to examine the fit of our data to the original and the proposed models. We performed the CFA using a robust estimator (Weighted Least Squares Mean and Variance adjusted, WLSMV), which is suitable for ordinal data and the non-normal distribution, typical of Likert scales. Model fit was evaluated with the following indices: chi-square (χ^2^), degree of freedom (df), root mean square error of approximation (RMSEA, <0.06), Comparative Fit Index (CFI, >0.95), Tucker-Lewis Index (TLI, >0.95) (29). After the CFA of the original three-factor model revealed poor fit indices, we proceeded with an Exploratory Factor Analysis (EFA). To determine the number of factors to extract, we considered both Kaiser’s criterion (eigenvalues above 1) and parallel analysis, which compares the actual eigenvalues from the data matrix to those generated from random datasets of identical size and structure (30). Prior to the EFA, Bartlett’s Test of Sphericity was applied to ensure the non-randomness of the correlation matrix (p-value should be <0.05), while the Kaiser-Meyer-Olkin (KMO) measure of sampling adequacy was calculated to ensure that the matrices were suitable for the analysis (should be >0.60) (31). We employed the unweighted least squares method with geomin oblique rotation (32). We assessed model-based reliability using coefficient omega hierarchical (OmegaH), explained common variance (ECV), and the percent of uncontaminated correlations (PUC). These indices help confirm that the total and subscale scores accurately reflect the intended constructs. Although there is no universally accepted cut-off for ωH, we followed Reise et al.’s recommendation that values above 0.50 indicate acceptable reliability, with values above 0.75 considered ideal (33). Furthermore, when PUC exceeds 0.80, the influence of general ECV values on bias is reduced. In cases where PUC is below 0.80, ECV values over 0.60 combined with ωH above 0.70 suggest that multidimensionality is not sufficiently strong to prevent interpreting the instrument as essentially unidimensional (33). For internal consistency measures, Cronbach’s α coefficients were calculated for the correlated factor models, in which 0.70-0.95 is the acceptable range (34). For test-retest reliability, the intraclass correlation coefficient (ICC) was computed along with the 95% confidence intervals based on a mean-rating (k=2), absolute-agreement, two-way mixed-effects model. The intraclass correlation coefficient (ICC) values were interpreted based on established guidelines (<0.50=poor; 0.50–0.75=moderate; 0.75–0.90=good; >0.90=excellent reliability) (35). To assess convergent validity, we used Pearson’s correlation, as the total scores of both the OMS-HC versions and the MICA scale followed a normal distribution. Intercorrelations between the specific factors and the general factor were examined using either Spearman’s or Pearson’s correlation coefficients, depending on the distribution of the variables. Correlation strength was interpreted as follows: *r=*0.00–0.1 (negligible), 0.10–0.39 (weak), 0.40–0.69 (moderate), 0.70–0.89 (strong), and 0.90–1.00 (very strong) (36). Given its suitability for small to moderate sample sizes, the Shapiro–Wilk test was employed to assess normality. Statistical analyses were conducted using IBM SPSS version 26.0.0.0 (Apache Software Foundation, USA) and Mplus version 8.0 (Muthén & Muthén, USA).

## 3 Results

### 3.1 Participants

Altogether, there were *n=*328 survey completions across two rounds for the assessment of test–retest reliability, from which *n=*48 responses could be successfully paired using the provided pseudonyms. However, as the time intervals between completions varied considerably, participants with intervals above the third quartile or below the first quartile of the interquartile range were excluded to minimize potential bias from extreme values. This resulted in a final test–retest sample of n=26 participants, with a median interval of 48 days (IQR=26.75–57.75 days) between the two completions. A total of 280 participants completed the survey. The sample comprised diverse healthcare professionals, including psychologists (n=97, 34.6%), psychiatrists (n=65, 23.2%), medical students (n=59, 21.1%), medical nurses (n=34, 12.1%), and other healthcare providers such as occupational therapists, special needs teachers, and social workers (n=25, 8.9%). Participants were predominantly in the 24–35-year age group (n=130, 46.4%), followed by those aged 36–45 years (n=80, 28.6%) and under 24 years (n=28, 10.0%). The remaining respondents were distributed across older age categories. The vast majority of the sample identified as female (n=240, 85.7%), while n=35 participants (12.5%) identified as male, and 5 (1.8%) selected “other” or preferred not to disclose their gender, reflecting the predominantly female composition of the Slovenian healthcare workforce.

### 3.2 Confirmatory factor analysis and model refinement using exploratory factor analysis

**Figure 1:**
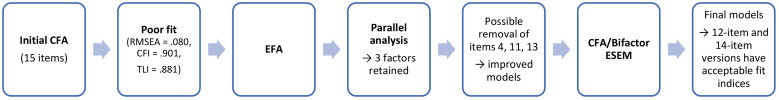
Flowchart summarizing the factor-analytic decisions for the Slovenian OMS-HC scale. CFA, confirmatory factor analysis; RMSEA, root mean square error of approximation; CFI, comparative fit index; TLI, Tucker-Lewis index; EFA, exploratory factor analysis; ESEM, exploratory structural equation modelling.

To illustrate the flow of factor analysis decisions, see **Figure 1**. The initial CFA of the original three correlated factor model with 15 items demonstrated a poor fit, with an RMSEA of 0.080 (90% CI: 0.070–0.089), and suboptimal comparative fit index (CFI=0.901) and Tucker-Lewis index (TLI=0.881). For all CFA results, see **Table 1**. While some authors consider RMSEA < 0.08 acceptable, others recommend a stricter cutoff of 0.06, and CFI and TLI > 0.90 are generally regarded as indicators of good model fit. In our model, the RMSEA value of 0.080 was at the upper limit of acceptability, the CFI reached the acceptable threshold, while the TLI was slightly below it. Given these borderline fit indices, we decided to conduct an EFA to further examine the factor structure of the Slovenian version of the scale and identify the model that best fits (For EFA results, see **Table 2**).

**Table 1:** Results of the series of confirmatory factor analyses.

Model	Chi-square	df	RMSEA (90% CI)	CFI	TLI	WRMR
3 correlated factors with 15 items	316.946	87	0.080 (0.070 - 0.089)	0.901	0.881	1.295
3 correlated factors with 15 items based on the EFA results	239.737	87	0.065 (0.055 - 0.075)	0.885	0.862	1.091
3 correlated factors with 14 items based on the EFA results	181.094	74	0.059 (0.048 – 0.070)	0.914	0.895	0.983
3 correlated factors with 12 items	128.554	51	0.060 (0.047 -0.073)	0.931	0.911	0.940
Bifactor with 15 items	241.325	75	0.073 (0.063 - 0.083)	0.929	0.900	1.068
Bifactor with 14 items based on the EFA results	150.278	63	0.0058 (0.046 – 0.070)	0.958	0.940	0.861
Bifactor with 12 items	99.348	42	0.057 (0.043 - 0.072)	0.967	0.949	0.784
Bifactor ESEM with 15 items	104.489	51	0.050 (0.036 - 0.064)	0.977	0.953	0.565
Bifactor ESEM with 14 items based on the EFA results	81.829	41	0.049 (0.033 – 0.064)	0.981	0.957	0.538
Bifactor ESEM with 12 items	31.273	24	0.027 (0.000 -0.051)	0.996	0.989	0.363

df, degrees of freedom; RMSEA, root mean square error of approximation; 90% CI, 90% confidence interval; CFI, comparative fit index; TLI, Tucker-Lewis index; WRMR, weighted root mean square residual.

**Table 2:** Results of the exploratory factor analysis.

Item	Original factor	First factor (disclosure and help-seeking)	Second factor (social distance)	Third factor (attitude)
1 I am more comfortable helping a person who has a physical illness than I am helping a person who has a mental illness.	Attitude	0.045	0.023	**0.553**
2 If a colleague with whom I work told me they had a managed mental illness, I would be as willing to work with him/her.	Social distance	0.051	**0.511**	0.096
3 If I were under treatment for a mental illness, I would not disclose this to any of my colleagues.	Disclosure and help-seeking	**0.695**	0.239	0.014
4 I would see myself as weak if I had a mental illness and could not fix it myself.	Disclosure and help-seeking	**0.412**	0.000	**0.443**
5 I would be reluctant to seek help if I had a mental illness.	Disclosure and help-seeking	**0.505**	-0.026	0.231
6 Employers should hire a person with a managed mental illness if he/she is the best person for the job.	Social distance	0.022	**0.482**	0.024
7 I would still go to a physician if I knew that the physician had been treated for a mental illness.	Social distance	0.039	**0.703**	-0.044
8 If I had a mental illness, I would tell my friends.	Disclosure and help-seeking	**0.513**	**0.363**	-0.083
9 Despite my professional beliefs, I have negative reactions towards people who have mental illness.	Attitude	0.057	0.096	**0.590**
10 There is little I can do to help people with mental illness.	Attitude	0.031	-0.030	**0.629**
11 More than half of people with mental illness don’t try hard enough to get better.	Attitude	-0.016	**0.359**	0.264
12 I would not want a person with a mental illness, even if it were appropriately managed, to work with children.	Social distance	-0.078	**0.520**	0.230
13 Health care providers do not need to be advocates for people with mental illness	Attitude	0.088	**0.394**	0.030
14 I would not mind if a person with a mental illness lived next door to me.	Social distance	-0.017	**0.387**	0.192
15 I struggle to feel compassion for a person with a mental illness.	Attitude	-0.026	0.197	**0.503**

Factor loadings above 0.3 are indicated in bold.

The KMO measure was 0.817, and Bartlett’s Test of Sphericity was significant (p < 0.0001), indicating that the data were suitable for factor analysis. Four eigenvalues exceeded one (3.842, 1.727, 1.450, and 1.014), indicating a four-factor solution according to the Kaiser criterion. However, parallel analysis — considered the gold standard for the determination of the number of factors to be retained —suggested the extraction of three factors. Given its alignment with Modgill’s conceptualization (16), we proceeded with extracting three factors. Geomin-rotated loadings revealed that item 4 exhibited severe cross-loading on the first and third factors (0.412 vs. 0.443), indicating poor discrimination and supporting its removal. Item 11 also showed cross-loading—though less pronounced—on the third factor. Additionally, item 13 loaded primarily onto a different factor than expected. Specifically, both items 11 and 13 showed stronger associations with the social distance factor rather than the intended attitude factor. These deviations suggested a misalignment with the Canadian results and theoretical structure, indicating the potential removal of these items from subsequent analyses.

**Figure 2:**
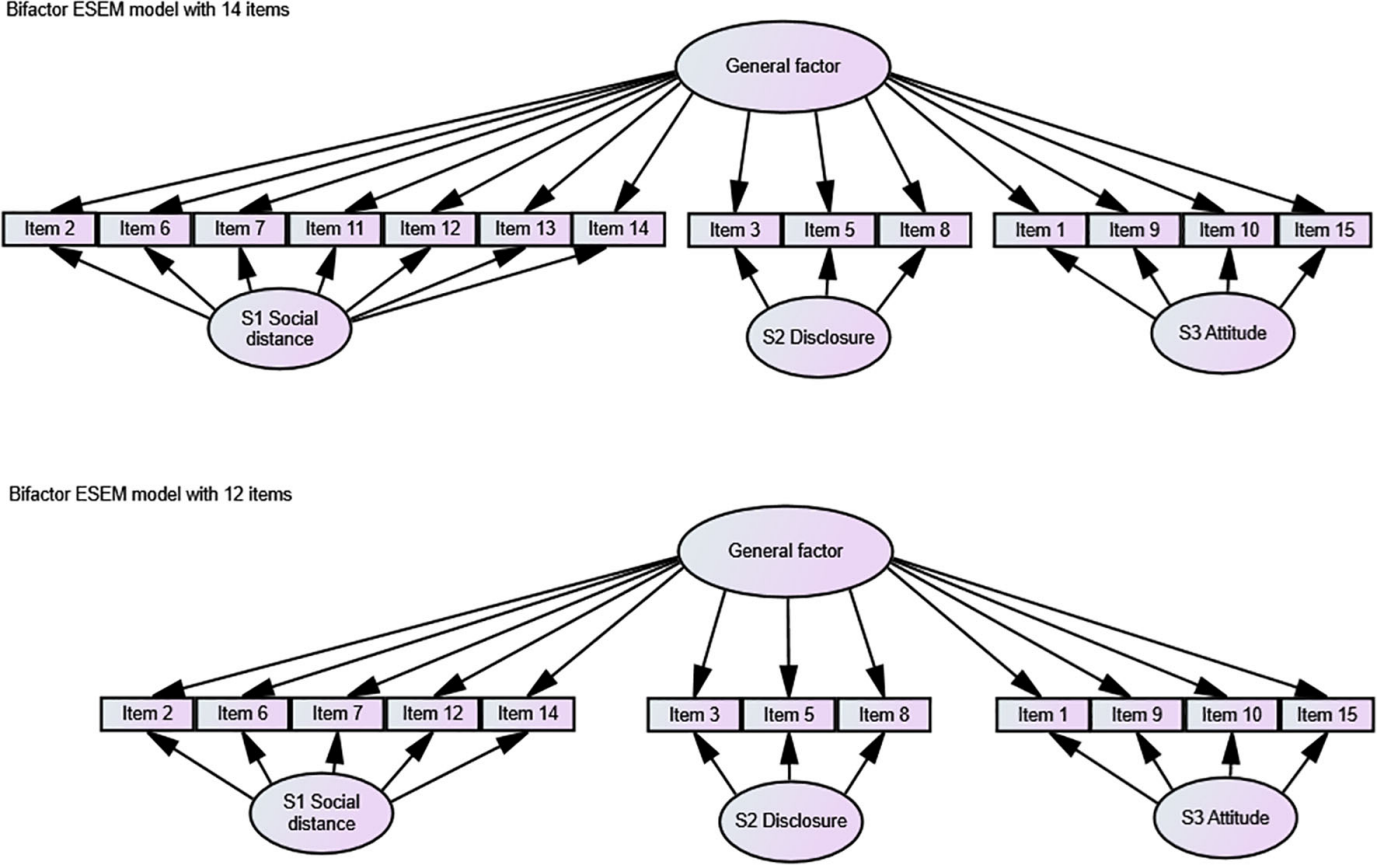
Final bifactor solutions.

In response, we explored whether removing problematic items could improve model fit. First, we eliminated item 4 to test the fit indices of the correlated factor model; however, the TLI of the relative fit indices did not meet the acceptable criteria. The elimination of all problematic items (4, 11, and 13) led to an improved model, with fit indices just falling within acceptable ranges (RMSEA=0.060, CFI=0.931, TLI=0.911). To ensure comparability of the Slovenian model fit results with other international studies on the OMS-HC scale, we also conducted CFA for bifactor models with 15, 14, and 12 items, as well as the bifactor ESEM approach with 15, 14, and 12 items, which has been shown to better account for item complexity. For the final bifactor solutions, please see **Figure 2**.

The 12-item and 14-item versions demonstrated better model fit than the 15-item versions across all models. However, the bifactor ESEM model with 15 items still produced fit indices within predefined acceptable ranges.

Taken together, our findings suggest that the Slovene version of the OMS-HC scale should consist of either 14 items—acknowledging that two items load on different factors than in the revised version proposed by Modgill et al. (16)—or 12 items, in which case all problematic items are removed. The correlated factor structure appears suitable, with three subscales: Attitude (items 1, 9, 10, 15), Disclosure and Help-Seeking (items 3, 5, 8), and Social Distance (items 2, 6, 7, 12, 14). Additionally, incorporating a general factor, the bifactor and bifactor ESEM approaches also provided good fit indices, making them viable alternative factor solutions. All factor analyses were conducted on the full dataset. Because of the strong gender imbalance in the sample (85.7% female), the results should be interpreted with this demographic context in mind.

### 3.3 Reliability measures

#### 3.3.1 Model-based reliability

We evaluated model-based reliability of both the 12-item and 14-item models to determine which is more reliable. Model-based reliability indices for the 12- and 14-item BESEM models are presented in **Table 3**. In both models, the general factor (which refers to the total score) accounted for a substantial portion of the shared variance among items, with an Explained Common Variance (ECV) of 0.588 for the 12-item model and 0.608 for the 14-item model, indicating that approximately 60% of the common variance was attributable to the general factor. Omega values for the general factor were similarly high (0.857 and 0.865) in both models, and Omega hierarchical (OmegaH) values were 0.698 and 0.752, respectively, suggesting that a large proportion of the total score variance can be attributed to the general factor after accounting for specific dimensions. The Percent of Uncontaminated Correlations (PUC) values were also high and comparable across the two models (0.712 vs. 0.703).

**Table 3:** Model-based reliability indices of the 12- and 14-item ESEM models.

Dimension	12-item model				14-item model			
	ECV	Omega	OmegaH	PUC	ECV	Omega	OmegaH	PUC
General Factor	0.588	0.857	0.698	0.712	0.608	0.865	0.752	0.703
Specific Factor 1 (Attitude)	0.256	0.787	0.199		0.596	0.699	0.417	
Specific Factor 2 (Disclosure and help-seeking)	0.927	0.640	0.601		0.804	0.663	0.548	
Specific Factor 3 (Social distance)	0.273	0.792	0.180		0.191	0.815	0.089	

ECV, the explained common variance, OmegaH, coefficient omega hierarchical, PUC, percent of uncontaminated correlations.

Regarding the specific factors, the “Attitude” dimension showed an ECV of 0.256 in the 12-item model and 0.596 in the 14-item model. Omega values were 0.787 and 0.699, while OmegaH values were 0.199 and 0.417, respectively, indicating that the “Attitude” factor was more clearly defined in the 14-item version. The “Disclosure and Help-Seeking” factor demonstrated high ECVs in both models (0.927 in the 12-item and 0.804 in the 14-item), with Omega values of 0.640 and 0.663, and OmegaH values of 0.601 and 0.548, suggesting consistent and meaningful contributions to the total score. The “Social Distance” factor exhibited lower distinctiveness, with ECVs of 0.273 and 0.191, Omega values of 0.792 and 0.815, and low OmegaH values of 0.180 and 0.089, respectively.

#### 3.3.2 Test-retest reliability and internal consistency

Test–retest reliability and internal consistency results are summarized in **Table 4**. Cronbach’s alpha values for the total scores and the three subscales indicated good internal consistency in both versions. Test–retest reliability was first assessed in a sample of 48 participants over a median interval of 39 days (IQR=21.25–69), with the range spanning from 6 to 123 days between the two completions. The wide variability in test–retest intervals was likely attributable to the survey distribution method. To minimize the potential impact of extreme interval lengths on reliability estimates, participants with test–retest intervals falling outside the interquartile range (i.e., below the first or above the third quartile on the boxplot) were excluded. This procedure reduced the sample size from n=48 to n=26 participants and yielded a subsample with greater temporal consistency between survey completions (median interval=48 days, IQR=26.75–57.75 days). The total score of both the 12-item and 14-item versions demonstrated good reliability (ICC=0.755 and ICC=0.752), with Cronbach’s alpha values of 0.858 and 0.855, respectively. The Attitude and Disclosure and Help-Seeking subscales were identical across both models; both exhibited moderate reliability (ICC=0.725, ICC=0.677, respectively). The Social Distance subscale, which included two additional items in the 14-item version, achieved good reliability in both the 12 and 14-item versions (ICC=0.799, ICC=0.768, respectively).

**Table 4:** Test-retest reliability and internal consistency results for both versions.

Dimension	12-item version		14-item version		Interpretation of ICCs
	ICC (95%CI of ICC)	Cronbach’s alpha	ICC (95%CI of ICC)	Cronbach’s alpha	
Total score	0.755 (0.526-0.882)	0.858	0.752 (0.520-0.881)	0.855	Good
Attitudes	0.725 (0.475-0.867)	0.837	0.725 (0.475-0.867)	0.837	Moderate
Disclosure and help-seeking	0.677 (0.406-0.840)	0.810	0.677 (0.406-0.840)	0.810	Moderate
Social distance	0.799 (0.559-0.910)	0.906	0.768 (0.50-0.889)	0.875	Good to excellent

ICC, intraclass correlation coefficient, 95% CI, 95% confidence intervals. Absolute-agreement, two-way mixed-effects model, single measures.

### 3.4 Convergent validity

The Shapiro–Wilk test was not significant for either the 14-item version (p=0.139) or the 12-item one (p=0.220), suggesting that the assumption of normality was met. Pearson’s correlation coefficient indicated a moderate positive correlation between the MICA and OMS-HC total scores for both the 14-item (r=0.639) and 12-item versions (r=0.608).

### 3.5 Intercorrelations between subscales

We used Spearman’s correlation coefficients to assess the relationships between the total scores and the subscale scores for both the 14-item and 12-item versions of the scale. All correlations were statistically significant (p < 0.0001). For the 14-item version, the total score showed strong correlations with the Attitude subscale (r=0.773), the Disclosure and Help-Seeking subscale (r=0.607), and the Social Distance subscale (r=0.825). The Attitude subscale correlated moderately with Social Distance (r=0.450) and weakly with Disclosure and Help-Seeking (r=0.267). The correlation between Disclosure and Help-Seeking and Social Distance was also weak (r=0.246). In the 12-item version, the total score similarly showed strong correlations with Attitude (r=0.759), Disclosure and Help-Seeking (r=0.634), and Social Distance (r=0.759). The Attitude subscale again correlated weakly with both Social Distance (r=0.267) and Disclosure and Help-Seeking (r=0.267), while Disclosure and Help-Seeking showed a weak correlation with Social Distance (r=0.210).

## 4 Discussion

In this study, we aimed to examine the psychometric properties of the Slovene version of the OMS-HC using a relatively large, diverse sample of healthcare professionals. First, we reviewed international studies that employed the OMS-HC and tested whether the Slovenian version aligned with the revised model proposed by Modgill et al (16).

The fit indices of the correlated three-factor model did not meet the standard acceptable criteria for acceptable model fit (RMSEA was 0.080, TLI was 0.881). Consequently, we conducted an exploratory factor analysis (EFA) to examine how individual items loaded onto specific factors and to identify problematic items — those that either failed to load meaningfully or demonstrated significant cross-loadings.

Item 4 (“I would see myself as weak if I had a mental illness and could not fix it myself.”), originally associated with the Disclosure and Help-Seeking factor, exhibited strong cross-loading with the Attitude factor (0.412 vs. 0.443), suggesting poor discrimination and supporting its removal. Items 11 (“More than half of people with mental illness don’t try hard enough to get better.”) and 13 (“Health care providers do not need to be advocates for people with mental illness.”) also displayed substantial cross-loadings, aligning more closely with the Social Distance factor rather than their intended Attitude factor. These misalignments indicated a need to consider removing these items from further analyses. This raised the question of whether to exclude only item 4 and retain a 14-item version or to remove items 4, 11, and 13 and test a more concise 12-item version. Therefore, we evaluated the model fit of all candidate versions through CFA and then tested the reliability of both versions.

Recognizing the limitations of the correlated three-factor model, we tested a bifactor model for the 15, 14, and 12-item versions of the scale. The 15-item yielded improved but still suboptimal fit indices compared to the correlated model. Both the 14- and 12-item versions demonstrated acceptable fit, with the 12-item version showing slightly better fit indices. To better account for item complexity and to be able to compare results with studies of other nations, we also employed the bifactor ESEM approach. All three bifactor ESEM models (15-, 14-, and 12-item) demonstrated acceptable fit. Among all tested models—including correlated, bifactor, and bifactor ESEM structures—the bifactor ESEM approach provided the most accurate representation of the scale’s structure in the Slovenian context based on the fit indices. These results are consistent with findings from the broader European validation study (25).

While all bifactor ESEM versions are psychometrically acceptable, the results from EFA supported the use of the 12- or 14-item versions due to the problematic factor loading. The 12-item version demonstrated slightly superior model fit, while the 14-item version retained greater content coverage, with the removal of only one item. However, this broader coverage comes with a trade-off: two retained items (11 and 13) loaded onto different factors than theoretically expected, raising concerns about their conceptual alignment.

Cross-national comparisons further support the removal of problematic items. For example, in the Singaporean study (18), item 1 was removed due to low loadings, while item 12 demonstrated cross-loading. The Hungarian version (20) necessitated the removal of item 11 and two other items (items 13 and 14 according to the 15-item numbering) loaded on different factors. Similarly, in the German-Swiss study (21), items 11 and 13 showed significant cross-loadings, with item 14 loading on all three factors. These consistent issues with items 11 and 13 across multiple countries suggest that these items may present certain challenges, potentially due to their strongly evaluative wording, possible cultural incongruence, or subtle differences in how such statements are interpreted across varying health systems, social norms, or linguistic contexts. It is important to note that our sample was predominantly female (85.7%), which likely reflects the actual gender distribution in the Slovenian healthcare workforce. In Slovenia, over 85% of nursing staff and the majority of psychology and allied health professionals are women (37, 38), consistent with broader European trends in healthcare. Moreover, previous studies indicate that women—particularly those in healthcare—are more likely to participate in research and respond to online surveys than men (39). Consequently, the factor analysis results may primarily represent female rather than male perspectives.

We also evaluated model-based reliability indices for both the 12- and 14-item bifactor ESEM models. Both versions exhibited strong general factors, as indicated by high ECV and omega coefficients. Notably, the 14-item model demonstrated slightly higher OmegaH and ECV values for the general factor, suggesting a possible superior representation of a unidimensional construct. Furthermore, this model showed clearer differentiation within the Attitude subscale, indicating higher subscale reliability. The Disclosure and Help-Seeking factor maintained consistently high ECV across models but exhibited substantial shared variance with the general factor, while the Social Distance factor displayed lower OmegaH values, reflecting reduced specificity. These findings align with prior research, including Hungarian (20) and Brazilian (22) studies that explored model-based reliability within bifactor frameworks. Additionally, the large-scale European study involving 29 countries tested a common overall model-based reliability and emphasized the support for the use of the total score over individual subscales (25).

Test-retest reliability showed good stability for the total score and for the Attitude and Social Distance subscales. The Disclosure and Help-Seeking subscale demonstrated only moderate stability, which may indicate this dimension fluctuates over time. These findings are consistent with prior studies: test-retest reliability was excellent in Italy (1-week follow-up) (17), near-satisfactory in Canada (20-item version) (15), good-to-excellent in Hungary (1-month follow-up) (20), and moderate in Portugal (24).

For convergent validity, the MICA-4 scale has been widely used internationally. Studies in Hungary (20), Brazil (22), and Chile (19) showed moderate to good correlations between the OMS-HC and MICA.

This study has several limitations. First, due to the absence of a validated Slovenian version of a comparable stigma scale, convergent validity was assessed using the MICA-4 scale, which cannot be considered a definitive gold standard, as it has not been validated locally; therefore, criterion validity could not be fully established. Second, while structural validity was evaluated, other important forms of validity—such as predictive validity, discriminant validity, and face validity—were not assessed in this study. Future research should test these additional forms of validity. Third, as this is a self-reported measure, it carries the potential for social desirability bias, which may limit its accuracy compared to more objective methods, such as behavioral observations or implicit stigma assessments. Fourth, there is a strong overrepresentation of female participants (85.7%, *n=*240), which is not uncommon in survey-based research and is consistent with the broader trend of female predominance in mental health and helping professions (e.g., psychologists, social workers, nurses) (40, 41). In Slovenia, women constitute the majority of healthcare workers—over 85% of nursing staff and most professionals in psychology and allied health fields (37, 38) —a trend consistent with broader European healthcare patterns, where female representation in clinical and mental health roles substantially exceeds that of men. Nonetheless, this imbalance should be acknowledged as a potential limitation, as the findings may not be fully representative of male professionals or reflect gender-specific perspectives in the field, which should be addressed in future studies. Due to the small number of male respondents, separate sensitivity analyses or gender-stratified factor analyses were not feasible. Lastly, the sample may not be fully representative of the broader population of Slovenian healthcare professionals, as participants were recruited from a limited number of institutions — approximately 80% of child psychiatry institutions and 50% of adult psychiatry institutions nationwide. Although the survey reached a large share of psychiatric hospitals and outpatient clinics and yielded responses from nurses and other allied mental health professionals, the absence of systematic national mailing lists likely reduced uniform exposure across nursing and occupational therapy settings, potentially affecting representativeness. This may limit the generalizability of the findings within the national context. An additional limitation is that general practitioners—who are typically in close contact with patients experiencing mental health conditions—were not included in this study. An important limitation is also the absence of direct input from service users or people with lived experience in the translation and cultural adaptation process—a reflection of the current lack of organizations or services facilitating such involvement in Slovenia. This limitation may affect face validity and cultural relevance and highlights the need for future developments in stakeholder engagement infrastructure.

Overall, the OMS-HC is a widely used instrument for assessing mental health-related stigma among healthcare professionals, capturing key dimensions such as attitudes toward individuals with mental illness, disclosure and help-seeking tendencies, and preferences for social distance. In Slovenia, no comparable validated scale exists to measure these constructs among healthcare providers, highlighting the significant need for such an instrument. Previous research in Slovenia has primarily focused on attitudes of mental health nurses toward adolescents engaging in non-suicidal self-injury (42) and on stigma within the general population, where prior help-seeking behavior was associated with reduced stigmatizing attitudes (43). Thus, the current validation of the OMS-HC addresses an important gap by introducing a psychometrically validated tool for measuring mental health-related stigma among Slovenian healthcare professionals, which is critical for designing anti-stigma initiatives and improving mental health care quality.

In conclusion, the validated Slovenian OMS-HC enables systematic assessment of mental health-related stigma among healthcare professionals at all levels of care. The 14-item version offers a stable and conceptually comprehensive structure, particularly when the total score is of interest and when broader content coverage—including advocacy and recovery beliefs—is valued. Alternatively, the 12-item version demonstrates a clearer factor structure and slightly superior model fit indices, making it a viable choice when model parsimony is prioritized. Both the 12 and 14-item versions are suitable for future research and practical implementation in Slovenian mental health care; however, we suggest the 12-item version for research studies prioritizing parsimony and the 14-item version when broader clinical coverage is desired. The implementation of OMS-HC scale could support several national priorities: (1) integration into training and continuing education programs to identify and address stigma among current and future providers; (2) evaluation of the effectiveness of anti-stigma interventions and policy initiatives by providing a standardized outcome measure; and (3) curriculum development for medical, nursing, and allied health education by identifying areas most in need of targeted stigma-reduction efforts. Regular use of the OMS-HC could thus facilitate monitoring, guide quality improvement, and help fulfil Slovenia’s commitment to enhancing mental health services in line with European best practices.

## Data Availability

The raw data supporting the conclusions of this article will be made available by the authors upon reasonable request to the corresponding author, Dorottya Őri (oridorottya@gmail.com).

## References

[1] Henderson C Noblett J Parke H Clement S Caffrey A Gale-Grant O . Mental health-related stigma in health care and mental health-care settings. Lancet Psychiatry. (2014) 1:467–82. doi: 10.1016/S2215-0366(14)00023-6, PMID: 26361202

[2] Perry A Lawrence V Henderson C . Stigmatisation of those with mental health conditions in the acute general hospital setting. A qualitative framework synthesis. Soc Sci Med. (2020) 255:112974. doi: 10.1016/j.socscimed.2020.112974, PMID: 32388323

[3] Thornicroft G . Stigma and discrimination limit access to mental health care. Epidemiol Psychiatr Sci. (2008) 17:14–9. doi: 10.1017/S1121189X00002621, PMID: 18444452

[4] Corrigan PW Rao D . On the self-stigma of mental illness: Stages, disclosure, and strategies for change. Can J Psychiatry. (2012) 57:464–9. doi: 10.1177/070674371205700804, PMID: 22854028 PMC3610943

[5] Valic M Knifton L Svab V . A review of the literature and media reports of patterns of mental health stigma in Slovenia until 2010. Slovenian J Public Health. (2013) 52:47–58. doi: 10.2478/sjph-2013-0006

[6] Možina M Okorn I . Challenges of the development of mental health care in Slovenia. J Global Health Neurol Psychiatry. (2022):e2022001. doi: 10.52872/001c.31788

[7] Thornicroft G Brohan E Rose D Sartorius N Leese M . Global pattern of experienced and anticipated discrimination against people with schizophrenia: a cross-sectional survey. Lancet. (2009) 373:408–15. doi: 10.1016/S0140-6736(08)61817-6, PMID: 19162314

[8] Mivšek AP Hundley V Kiger A . Slovenian midwives’ and nurses’ views on post-natal depression: an exploratory study. Int Nurs Review. (2008) 55:320–6. doi: 10.1111/j.1466-7657.2008.00620.x, PMID: 19522949

[9] Trobec I Herbst M Žvanut B . Differentiating between rights-based and relational ethical approaches. Nurs Ethics. (2009) 16:283–91. doi: 10.1177/0969733009102689, PMID: 19372123

[10] Uplaznik Š Vaupotič K Kumperščak HG Ilješ AP . Attitudes toward mental disorder among medical students. Zdrav Vestn. (2022) 91:273–84. doi: 10.6016/ZdravVestn.3311

[11] Vučinić N Holnthaner R Plakolm Erlač S Skokauskas N Gregorič Kumperščak H . Stigma about mental health in Slovenian first-year medical students. J Med Educ Curricular Dev. (2024) 11:23821205241283751. doi: 10.1177/23821205241283751, PMID: 39497904 PMC11533269

[12] Harhaji S Tomori S Nakov V Chihai J Radić I Mana T . Stigmatising attitudes towards mental health conditions among medical students in five South-Eastern European countries. Slovenian J Public Health. (2024) 63:188. doi: 10.2478/sjph-2024-0025, PMID: 39319025 PMC11417508

[13] Janoušková M Formánek T Bražinová A Mílek P Alexová A Winkler P . Attitudes towards People with Mental Illness and Low Interest in Psychiatry among Medical Students in Central and Eastern Europe. Psychiatr Q. (2021) 92:407–18. doi: 10.1007/s11126-020-09817-3, PMID: 32780288

[14] Őri D Szocsics P Molnár T Ralovich FV Huszár Z Bene Á . Stigma towards mental illness and help-seeking behaviors among adult and child psychiatrists in Hungary: A cross-sectional study. PloS One. (2022) 17:e0269802. doi: 10.1371/journal.pone.0269802, PMID: 35687584 PMC9187077

[15] Kassam A Papish A Modgill G Patten S . The development and psychometric properties of a new scale to measure mental illness related stigma by health care providers: the Opening Minds Scale for Health Care Providers (OMS-HC). BMC Psychiatry. (2012) 12:62. doi: 10.1186/1471-244X-12-62, PMID: 22694771 PMC3681304

[16] Modgill G Patten SB Knaak S Kassam A Szeto AC . Opening minds stigma scale for health care providers (OMS-HC): examination of psychometric properties and responsiveness. BMC Psychiatry. (2014) 14:120. doi: 10.1186/1471-244X-14-120, PMID: 24758158 PMC4024210

[17] Destrebecq A Ferrara P Frattini L Pittella F Rossano G Striano G . The Italian version of the opening minds stigma scale for healthcare providers: Validation and study on a sample of bachelor students. Community Ment Health J. (2018) 54:66–72. doi: 10.1007/s10597-017-0149-0, PMID: 28647819

[18] Chang S Ong HL Seow E Chua BY Abdin E Samari E . Stigma towards mental illness among medical and nursing students in Singapore: a cross-sectional study. BMJ Open. (2017) 7:e018099. doi: 10.1136/bmjopen-2017-018099, PMID: 29208617 PMC5719274

[19] Sapag JC Klabunde R Villarroel L Velasco PR Álvarez C Parra C . Validation of the Opening Minds Scale and patterns of stigma in Chilean primary health care. PloS One. (2019) 14. doi: 10.1371/journal.pone.0221825, PMID: 31487333 PMC6728029

[20] Őri D Rózsa S Szocsics P Simon L Purebl G Győrffy Z . Factor structure of The Opening Minds Stigma Scale for Health Care Providers and psychometric properties of its Hungarian version. BMC Psychiatry. (2020) 20:1–9. doi: 10.1186/s12888-020-02902-8, PMID: 33046048 PMC7552521

[21] Zuaboni G Elmer T Rabenschlag F Heumann K Jaeger S Kozel B . Psychometric evaluation of the German version of the Opening Minds Stigma Scale for Health Care Providers (OMS-HC). BMC Psychol. (2021) 9:1–7. doi: 10.1186/s40359-021-00592-9, PMID: 34016166 PMC8139058

[22] Carrara BS Sanches M Bobbili SJ de Godoy Costa S de Sousa ÁFL de Souza J . Validation of the opening minds scale for health care providers (OMS-HC): factor structure and psychometric properties of the Brazilian version. Healthcare. (2023) 11:1049. doi: 10.3390/healthcare11071049, PMID: 37046976 PMC10094058

[23] Ramos HNV Mora-Rios J Natera G Mondragón L . Psychometric properties of the Mexican version of the opening minds stigma scale for health care providers (OMS-HC). PeerJ. (2023) 11:e16375. doi: 10.7717/peerj.16375, PMID: 38025693 PMC10655721

[24] Moreira MBP Pereira HP Torres IN Marina S Ricou M . The stigma towards mental illness: Portuguese validation of the Opening Minds Stigma Scale for Healthcare Providers (OMS-HC). Front Psychol. (2024) 15:1359483. doi: 10.3389/fpsyg.2024.1359483, PMID: 38515965 PMC10955081

[25] Őri D Szocsics P Molnár T Bankovska Motlova L Kazakova O Mörkl S . Psychometric properties of the Opening Minds Stigma Scale for Health Care Providers in 32 European countries – A bifactor ESEM representation. Front Public Health. (2023) 11. doi: 10.3389/fpubh.2023.1168929, PMID: 37361150 PMC10285467

[26] Krohne N Gomboc V Lavrič M Podlogar T Poštuvan V Šedivy NZ . Slovenian validation of the mental health literacy scale (S-MHLS) on the general population: a four-factor model. INQUIRY: J Health Care Organization Provision Financing. (2022) 59:00469580211047193. doi: 10.1177/00469580211047193, PMID: 35135367 PMC8832589

[27] Őri D Szocsics P Molnár T Motlova LB Kazakova O Mörkl S . Attitudes of psychiatrists towards people with mental illness: a cross-sectional, multicentre study of stigma in 32 European countries. EClinicalMedicine. (2023) 66. doi: 10.1016/j.eclinm.2023.102342, PMID: 38149261 PMC10749877

[28] Gabbidon J Clement S van Nieuwenhuizen A Kassam A Brohan E Norman I . Mental Illness: Clinicians’ Attitudes (MICA) Scale—Psychometric properties of a version for healthcare students and professionals. Psychiatry Res. (2013) 206:81–7. doi: 10.1016/j.psychres.2012.09.028, PMID: 23084597

[29] Hu Lt Bentler PM . Cutoff criteria for fit indexes in covariance structure analysis: Conventional criteria versus new alternatives. Struct Equation Modeling: A Multidiscip J. (1999) 6:1–55. doi: 10.1080/10705519909540118

[30] Horn JL . A rationale and test for the number of factors in factor analysis. Psychometrika. (1965) 30:179–85. doi: 10.1007/BF02289447, PMID: 14306381

[31] Tabachnick BG Fidell LS Ullman JB . Principal components and factor analysis. Using multivariate statistics Vol. 5. . Boston, MA: Pearson Education (2007) p. 614–6.

[32] Schmid J Leiman JM . The development of hierarchical factor solutions. Psychometrika. (1957) 22:53–61. doi: 10.1007/BF02289209

[33] Reise SP Scheines R Widaman KF Haviland MG . Multidimensionality and structural coefficient bias in structural equation modeling:A bifactor perspective. Educ psychol Measurement. (2013) 73:5–26. doi: 10.1177/0013164412449831

[34] DeVellis RF . Scale development: Theory and applications. Thousand Oaks, CA: Sage publications (2016).

[35] Koo TK Li MY . A guideline of selecting and reporting intraclass correlation coefficients for reliability research. J Chiropr Med. (2016) 15:155–63. doi: 10.1016/j.jcm.2016.02.012, PMID: 27330520 PMC4913118

[36] Schober P Boer C Schwarte LA . Correlation coefficients: appropriate use and interpretation. Anesth Analgesia. (2018) 126:1763–8. doi: 10.1213/ANE.0000000000002864, PMID: 29481436

[37] Prosen M Čekada T . Nursing students’ views on men in nursing: a gender diversity challenge in the healthcare workforce. BMC nursing. (2025) 24:820. doi: 10.1186/s12912-025-03521-y, PMID: 40605023 PMC12219806

[38] Zajc JČ Hafner A . Gender differences in employee health in Slovenia: the role of work intensity, organisational commitment and mobbing. Družboslovne Razprave. (2020) 36:87–107.

[39] Kozjek T Erčulj VI . Mistreatment by patients: an analysis of the patient-related social stressors among Slovenian healthcare workers. Slovenian J Public Health. (2021) 60:90. doi: 10.2478/sjph-2021-0014, PMID: 33822829 PMC8015656

[40] Bubeck DE . Care, gender, and justice. Oxford, UK: Oxford University Press (1995).

[41] Dysvik E Sommerseth R . A man could never do what women can do: Mental health care and the significance of gender. Patient preference adherence. (2010), 77–86. doi: 10.2147/PPA.S9103, PMID: 20517468 PMC2875717

[42] Pintar Babič M Bregar B Drobnič Radobuljac M . The attitudes and feelings of mental health nurses towards adolescents and young adults with nonsuicidal self-injuring behaviors. Child Adolesc Psychiatry Ment Health. (2020) 14:1–10. doi: 10.1186/s13034-020-00343-5, PMID: 32973922 PMC7508242

[43] Roskar S Bracic MF Kolar U Lekic K Juricic NK Grum AT . Attitudes within the general population towards seeking professional help in cases of mental distress. Int J Soc Psychiatry. (2017) 63:614–21. doi: 10.1177/0020764017724819, PMID: 28795635

