# Supplementary material for: Psychometric properties and validation of the Opening Minds Stigma Scale for Health Care Providers in Slovenia
Source: Front Psychiatry. 2025 Nov 12;16:1671589. doi: 10.3389/fpsyt.2025.1671589 (PMC12650770; doi:10.3389/fpsyt.2025.1671589)
Supplement: Supplementary file 1 [file Table1.docx]

**Supplementary material**

**Slovenian version of the 15-, 14-, and 12-item Opening Minds Stigma Scale for Health Care Providers**

**Opening Minds Stigma Scale for Health Care Providers (Original, 15-item version)**

Prosimo preberite vsako od naslednjih trditev in ocenite v kolikšni meri opisuje vase občutke do ljudi z duševno boleznijo.

Prosimo izberite enega izmed naslednjih možnih odgovorov:

- Povsem se strinjam
- Se strinjam
- Sem neodločen
- Ne strinjam se
- Sploh se ne strinjam

| 1 | Bolj prijetno mi je pomagati osebi, ki ima telesno bolezen kot pomagati osebi, ki ima duševno bolezen. |
| --- | --- |
| 2 | Če bi mi sodelavec s katerim delam rekel, da ima pozdravljeno duševno bolezen, bi bil/a prav tako pripravljen z njim sodelovati. |
| 3 | Če bi bil/a na zdravljenju zaradi duševne bolezni, tega ne bi razkril/a nobenemu od svojih kolegov. |
| 4 | Imel/a bi se za šibkega/šibko, če bi imel/a duševno bolezen in je ne bi zmogel/la sam/a pozdraviti. |
| 5 | Če bi imel/a duševno bolezen, bi bil/a zadržan/a do iskanja pomoči. |
| 6 | Delodajalci bi morali zaposliti osebo z zazdravljeno duševno boleznijo, če je ta oseba najboljša za delovno mesto. |
| 7 | Še vedno bi šel/šla k zdravniku, če bi vedel/a, da se je zdravil zaradi duševne bolezni. |
| 8 | Če bi imel/a duševno bolezen, bi povedal/a prijateljem. |
| 9 | Kljub mojim poklicnim prepričanjem, imam negativne reakcije do ljudi, ki imajo duševno bolezen. |
| 10 | Malo lahko storim, da pomagam ljudem z duševno boleznijo. |
| 11 | Več kot polovica ljudi z duševno boleznijo se ne trudi dovolj, da bi se izboljšala. |
| 12 | Ne bi si želel/a, da bi oseba z duševno boleznijo, četudi bi bila ustrezno zazdravljena, delala z otroki. |
| 13 | Zdravstveni delavci ne rabijo biti zagovorniki ljudi z duševno boleznijo. |
| 14 | Ne bi imel/a nič proti, če bi imel/a za soseda osebo z duševno boleznijo. |
| 15 | S težavo čutim sočutje do osebe z duševno boleznijo. |

**Original subscales:**

Attitude: items 1,9,10,11,13,15

Disclosure and help-seeking: items 3,4,5,8

Social distance: items 2,6,7,12,14

**Opening Minds Stigma Scale for Health Care Providers (Slovenian 14-item version)**

Prosimo preberite vsako od naslednjih trditev in ocenite v kolikšni meri opisuje vase občutke do ljudi z duševno boleznijo.

Prosimo izberite enega izmed naslednjih možnih odgovorov:

- Povsem se strinjam
- Se strinjam
- Sem neodločen
- Ne strinjam se
- Sploh se ne strinjam

| New numbering | Original numbering | Statements |
| --- | --- | --- |
| 1 | 1 | Bolj prijetno mi je pomagati osebi, ki ima telesno bolezen kot pomagati osebi, ki ima duševno bolezen. |
| 2 | 2 | Če bi mi sodelavec s katerim delam rekel, da ima pozdravljeno duševno bolezen, bi bil/a prav tako pripravljen z njim sodelovati. |
| 3 | 3 | Če bi bil/a na zdravljenju zaradi duševne bolezni, tega ne bi razkril/a nobenemu od svojih kolegov. |
| 4 | 5 | Če bi imel/a duševno bolezen, bi bil/a zadržan/a do iskanja pomoči. |
| 5 | 6 | Delodajalci bi morali zaposliti osebo z zazdravljeno duševno boleznijo, če je ta oseba najboljša za delovno mesto. |
| 6 | 7 | Še vedno bi šel/šla k zdravniku, če bi vedel/a, da se je zdravil zaradi duševne bolezni. |
| 7 | 8 | Če bi imel/a duševno bolezen, bi povedal/a prijateljem. |
| 8 | 9 | Kljub mojim poklicnim prepričanjem, imam negativne reakcije do ljudi, ki imajo duševno bolezen. |
| 9 | 10 | Malo lahko storim, da pomagam ljudem z duševno boleznijo. |
| 10 | 11 | Več kot polovica ljudi z duševno boleznijo se ne trudi dovolj, da bi se izboljšala. |
| 11 | 12 | Ne bi si želel/a, da bi oseba z duševno boleznijo, četudi bi bila ustrezno zazdravljena, delala z otroki. |
| 12 | 13 | Zdravstveni delavci ne rabijo biti zagovorniki ljudi z duševno boleznijo. |
| 13 | 14 | Ne bi imel/a nič proti, če bi imel/a za soseda osebo z duševno boleznijo. |
| 14 | 15 | S težavo čutim sočutje do osebe z duševno boleznijo. |

(Item 4 was removed)

**14-item subscales using the original numbers:**

Attitude: items 1,9,10,15

Disclosure and help-seeking: items 3,5,8

Social distance: items 2,6,7,12,14, 11,13

**Opening Minds Stigma Scale for Health Care Providers (Slovenian 12-item version)**

Prosimo preberite vsako od naslednjih trditev in ocenite v kolikšni meri opisuje vase občutke do ljudi z duševno boleznijo.

Prosimo izberite enega izmed naslednjih možnih odgovorov:

- Povsem se strinjam
- Se strinjam
- Sem neodločen
- Ne strinjam se
- Sploh se ne strinjam

| New numbering | Original numbering | Statements |
| --- | --- | --- |
| 1 | 1 | Bolj prijetno mi je pomagati osebi, ki ima telesno bolezen kot pomagati osebi, ki ima duševno bolezen. |
| 2 | 2 | Če bi mi sodelavec s katerim delam rekel, da ima pozdravljeno duševno bolezen, bi bil/a prav tako pripravljen z njim sodelovati. |
| 3 | 3 | Če bi bil/a na zdravljenju zaradi duševne bolezni, tega ne bi razkril/a nobenemu od svojih kolegov. |
| 4 | 5 | Če bi imel/a duševno bolezen, bi bil/a zadržan/a do iskanja pomoči. |
| 5 | 6 | Delodajalci bi morali zaposliti osebo z zazdravljeno duševno boleznijo, če je ta oseba najboljša za delovno mesto. |
| 6 | 7 | Še vedno bi šel/šla k zdravniku, če bi vedel/a, da se je zdravil zaradi duševne bolezni. |
| 7 | 8 | Če bi imel/a duševno bolezen, bi povedal/a prijateljem. |
| 8 | 9 | Kljub mojim poklicnim prepričanjem, imam negativne reakcije do ljudi, ki imajo duševno bolezen. |
| 9 | 10 | Malo lahko storim, da pomagam ljudem z duševno boleznijo. |
| 10 | 12 | Ne bi si želel/a, da bi oseba z duševno boleznijo, četudi bi bila ustrezno zazdravljena, delala z otroki. |
| 11 | 14 | Ne bi imel/a nič proti, če bi imel/a za soseda osebo z duševno boleznijo. |
| 12 | 15 | S težavo čutim sočutje do osebe z duševno boleznijo. |

(Items 4, 11, and 13 were removed)

**12-item version subscales using the original numbers:**

Attitude: items 1,9,10,15

Disclosure and help-seeking: items 3,5,8

Social distance: items 2,6,7,12,14
